# Keratin 8 expression in head and neck epithelia

**DOI:** 10.1186/1471-2407-8-267

**Published:** 2008-09-22

**Authors:** Christoph Matthias, Brigitte Mack, Alexander Berghaus, Olivier Gires

**Affiliations:** 1 Department of Otorhinolaryngology, Head and Neck Surgery, University of Goettingen Medical School, Robert-Kochstr. 40, 37075 Göttingen, Germany; 2 Department of Otorhinolaryngology, Head and Neck Surgery, Grosshadern Medical Center, Ludwig-Maximilians-University of Munich, Marchioninistr. 15, 81377 Munich, Germany; 3 Clinical Cooperation Group Molecular Oncology, Helmholtz-Zentrum München, German Research Center for Environmental Health, and Head and Neck Research Dept. Ludwig-Maximilians-University of Munich, Germany

## Abstract

**Background:**

The intermediate filament forming protein keratin 8 (K8) is a tumour-associated antigen, which was shown to be over-expressed in a variety of malignancies. Here, we present a study of K8 expression in squamous epithelia of the head and neck area, including normal mucosa, hyperplastic and dysplastic leukoplakia, carcinomas of different sub-localisations, and lymph node metastases.

**Methods:**

K8 expression was assessed upon immunohistochemistry with specific antibodies in cryosections of primary tumours of the head and neck area.

**Results:**

K8 expression was characteristic of transformed tissue and marked early stages of disease, *i.e. *dysplastic oral leukoplakia, but not normal or hyperplastic epithelium. With the exception of carcinomas of the larynx and the tongue, K8 expression also strictly differentiated carcinomas from normal epithelium of the same origin. Furthermore, K8^high ^was characteristic of cells, which had detached from the sites of primary tumours and had been invading the surrounding tissue at the time point of surgery.

**Conclusion:**

K8 is an excellent marker for head and neck malignancies, which allows for early detection as well as for visualisation of potentially disseminating tumour cells *in vivo*.

## Background

Cytokeratin 8 (K8) is a structural protein, which forms intermediate filaments within the cytoplasm of simple epithelial cells [1] as a dimer with CK18 [2]. Along with other keratins, K8/CK18 generate a stabilizing framework, which is cell shape determining and allows cells to cope with mechanical stress. Cytokeratin filaments further on represent a mesh of "paths" on which signalling molecules, metabolites, and pathogens can travel the cell in an orientated fashion. The regulation of the localization of K8 within cells and polymerization into intermediate filaments is dependent upon its phosphorylation. Two main kinase families are instrumental in this context: the MAP kinase family member p38 [3] and PKC-ε related kinase [4]. Phosphorylation of K8 at serine in position 73 (Ser^73^) is mediated by p38 under stress such as orthovanadate treatment, and regulates keratin organization [5]. High p38 kinase activity correlated with the formation of keratin granules, while low p38 activity, *ergo *low K8 Ser^73 ^phosphorylation, was associated with a prevented disassembly of the filament network [5]. As a potential counter-regulator and eventually in order to balance the phosphorylation status of K8, the catalytic subunit of protein phosphatase 2A (PP2A) associates with and dephosphorylates K8 after hyposmotic stress [6]. However, dephosphorylation was site-specific and concerned Ser^431^, not Ser^73^. Additionally, K8 and CK18 hyperphosphorylation is a valuable marker for the progression of liver diseases such as non-cirrhotic hepatitis C infection or cirrhosis [7]. Disease associated mutations of K8 were reported for the case of cryptogenic liver diseases with single point mutations leading to the exchange of glycine at position 61 to a cysteine residue and of tyrosine^53 ^to a histidine [8,9]. Gly^61 ^→ Cys mutation was of major importance as it diminished the capacity of cells to reorganize keratin filament. Recently, Ku and colleagues reported on an animal model for the Gly^61 ^→ Cys mutation. In transgenic mice, this point mutant of K8 predisposed animals to liver injury along with a decreased Ser73 phosphorylation [10]. When ectopically expressed at the plasma membrane of carcinoma cells [11], K8 serves as a tissue-type plasminogen activator (tPA) [12-15] and might help tumour cells to remodel or invade surrounding tissue [16].

Generally speaking, K8 is believed to be involved in the process of carcinogenesis [17-21] and silencing of it resulted in sensitization for cisplatin [22]. We have isolated K8 as a tumour-associated antigen, which elicits a humoral response *in vivo *in patients suffering from carcinomas of the head and neck area [23]. A continuative study on the profile of K8-specific autoantibodies in healthy donors and patients revealed that autoantibody titers allowed to differentiate normal and diseased persons, but not to discriminate between cases of benign and malignant disease [24]. Normal squamous epithelium, which represents the great majority of epithelia of the head and neck and of malignancies thereof, is devoid of K8. *De novo *expression of K8 was observed for head and neck carcinomas, however in a small patients cohort [25]. Studies including larger numbers of patients with head and neck malignancies are to the best of our knowledge missing so far and therefore the topic of the present investigation.

Here, we present a comprehensive survey of K8 expression in normal mucosa, leukoplakia, head and neck squamous cell carcinomas (HNSCC), and lymph node metastases of the head and neck area. We have used immunohistochemistry on cryosections for this purpose as it allows thorough detection of K8 and, importantly, the assignment of staining to particular cell types within samples as opposed to RT-PCR or immunoblotting. K8 positivity was a hallmark of transformed epithelia where it correlated with early stages of carcinogenesis in dysplastic leukoplakia. K8 was also strongly over-expressed in the majority of HNSCC tested. Importantly, K8^high ^was a characteristic of disseminated tumour cells and in overt lymph node metastases. For this reason, K8 represents a valuable marker for head and neck malignancies in early stages of the disease and of invasive growth of tumour cells.

## Methods

### Tissue samples

All human samples were obtained after informed consent according to the Helsinki Declaration and on the basis of an approval by the local ethical committee (Ethikkommision der Medizinischen Fakultät der Ludwig-Maximilians-Universität München; file reference N° 087/03). Fifty-seven samples of normal mucosa were obtained from patients suffering from chronic tonsillitis (n = 9), tonsilar carcinomas (n = 10), laryngeal carcinomas (n = 9), oropharyngeal carcinomas (n = 6), hypopharyngeal carcinomas (n = 4), carcinomas of the tongue (n = 7), mouth (n = 4), valeculla (n = 2), vocal cords (n = 2), nasopharynx (n = 1), alveolar ridge (n = 1), uvula (n = 1), and nasal concha hyperplasia (n = 1). Nineteen samples of oral leukoplakia, six samples of lymph node metastases, and one hundred and two samples of head and neck carcinomas of different localisation were collected. All specimens had been confirmed by routine clinical diagnosis according to the UICC TNM classification of 2003 for head and neck carcinomas [26] and histopathologic examination by two expert pathologists. Characteristics used for the diagnosis were tumour size (T), locoregional lymph node status (N), and the presence of distant metastases at the time of first diagnosis (M). Tissue specimen were shock-frozen in liquid nitrogen and embedded in tissue-tek (Sakura, Fintek, NL) to generate 4 μm non-consecutive sections.

### Immunohistochemistry

The mouse anti-human K8 clone 35βH11 primary antibody was used (Dako, Glostrup, DK), (diluted 1:100). Immunostaining was performed using the avidin-biotin-peroxidase complex method (Vectastain, Vector laboratories, Burlingame, CA, USA) according to the manufacturer's protocol. Briefly, after fixation in acetone (10 min), endogenous peroxidase activity was inhibited upon treatment with 0.03% H2O2/PBS (10 min). Before specific staining, unspecific antigenic sites were blocked with normal goat serum or normal horse serum. Sections were then incubated with the respective primary antibody for 1 hour at room temperature (RT) followed by incubation with biotinylated anti-rabbit or anti-mouse immunoglobulins and then with avidin-biotin-peroxidase complex (30 minutes at RT for each step). After each step, sections were washed with PBS. Specific peroxidase activity was visualized with 0.05% 3-amino-9-ethylcarbazol as a substrate (Sigma, Deisenhofen, Germany) and 0.02% H2O2/0,1 M Na-acetat buffer pH5.5). Counterstaining was performed with Mayers hematoxylin. Control staining was performed in the absence of primary antibody. Immunostained sections were evaluated upon light microscopy by two independent investigators. Double immunostainings were performed with a monoclonal anti Ki67 antibody (Dako, Glostrup, DK) using the avidin-biotin-peroxidase method (ABC, red-brown staining), together with the K8-specific 35βH11 antibody using alkaline phosphatase-anti-alkaline phosphatase method and fast Blue BB salt (Sigma, Deisenhofen, Germany) as a chromogenic substrate (deep blue staining). Negative controls were conducted in the absence of primary antibodies for every detection system. Sample evaluation was performed by two experienced researchers (BM and OG) according to criteria of staining intensities (0-+++) published elsewhere [25].

## Results

### K8 expression in head and neck epithelia

**Figure 1:**
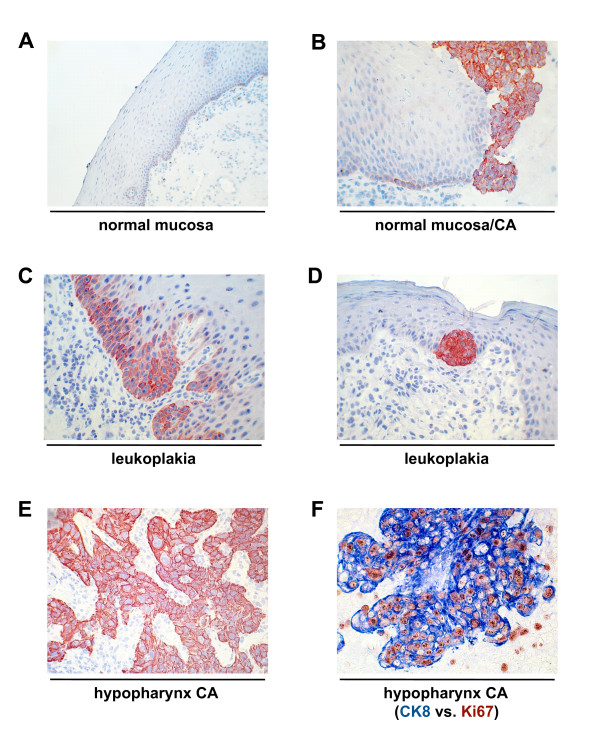
**K8 expression pattern in head and neck epithelia.** K8 was visualised by immunohistochemistry with specific antibodies in normal mucosa (**A**), hypopharynx carcinoma with adjacent normal mucosa (**B**), oral leukoplakia (**C, D**), hypopharynx carcinoma (**E, F**). Ki67 was stained in parallel to detect proliferating cells (**F**). Colour coding is indicated below figure 1F.

In a first step, we studied the expression pattern of K8 in a small subset of tissues including normal epithelium of the oropharynx, oral leukoplakia, and hypopharynx carcinomas. Appraisal of tissue integrity was performed after hematoxilin staining, while diagnosis was conducted beforehand by two expert pathologists according to UICC's TNM classification. Evaluation of K8 expression was subdivided into negative, weak, intermediate, and strong as was described in Gires *et al. *[25]. K8 was not detected in normal mucosa except for a very mild and incidental expression in cells of the *stratum basale *(Figure 1A). In a hypopharynx carcinoma specimen, both microscopically normal mucosa and malignant cells were adjacent. K8 staining allowed for a strict differentiation of both tissues with carcinoma cells expressing high levels of K8 while normal mucosa retained its typical staining pattern restricted to some cells of the *stratum basale *(Figure 1B). Staining of additional leukoplakia specimens demonstrated that K8 *de novo *expression is a fairly early event in cell transformation, which starts in cells of the basal membrane layer. In case regular epithelial stratification was lost and cells of the basal membrane layer proliferated to generate multiple layers, K8 expression was strongest in these cells and progressed to suprabasal cells (Figure 1C). Where incipient transformation was detected, this aberrant K8 synthesis could even be restricted to nests of cells which proliferated, protruded in the surrounding tissue, and lay in areas characterized by massive cellular infiltrates (Figure 1D and data not shown). In hypopharynx carcinomas the expression of K8 was almost in 100% of tumour cells and mostly strong (Figure 1E), and positively correlated with the marker of proliferation Ki67 (Figure 1F).

### K8 expression in diverse head and neck malignancies

Since increased or even *de novo *expression of K8 was characteristic of transformation in a small cohort of patients (data herein and Gires et al. 2006), we next studied K8 in a larger collective of patients. Firstly, microscopically normal mucosas (n = 57) were assessed. Tissue specimens originated from the oropharynx and larynx of patients suffering from either chronic tonsillitis, tonsillar carcinomas, laryngeal carcinomas, oropharyngeal carcinomas, hypopharyngeal carcinomas, tongue carcinomas, buccal carcinomas, carcinomas of the valeculla, vocal cords, nasopharynx, alveolar ridge, uvula, or from nasal concha hyperplasia. Cryosections were stained with hematoxillin in order to appreciate tissue integrity and non-transformed phenotypes. The second parameter was considered as a second appraisal following routine diagnosis by pathologists. Except for four oropharyngeal mucosas from tonsillar carcinoma (n = 1), oropharyngeal carcinomas (n = 2), and carcinoma of the valeculla (n = 1), 100% of oropharyngeal normal mucosas were devoid of K8 (Table 1). In all four divergent cases, K8 was expressed to intermediate (++) or strong (+++) levels in varying percentages of tumour cells included in the specimens. In sharp contrast, two laryngeal normal mucosas were strongly (+++) positive in 100% of cells (Table 1). In summary, normal oropharyngeal but not laryngeal mucosa was to 100% devoid of K8.

**Table 1:** K8 expression in normal mucosa.

**PATIENT**	**K8 INTENSITY**	**% OF TISSUE**	**LOCALISATION OF MUCOSA**
chron. Tonsillitis	-	/	oropharynx
chron. Tonsillitis	-	/	oropharynx
chron. Tonsillitis	-	/	oropharynx
chron. Tonsillitis	-	/	oropharynx
chron. Tonsillitis	-	/	oropharynx
chron. Tonsillitis	-	/	oropharynx
chron. Tonsillitis	-	/	oropharynx
chron. Laryngitis	-	/	oropharynx
chron. Tonsillitis	-	/	oropharynx
Ton Ca	+++	80	oropharynx *
Ton Ca	-	/	oropharynx
Ton Ca	-	/	oropharynx
Ton Ca	-	/	oropharynx
Ton Ca	-	/	oropharynx
Ton Ca	-	/	oropharynx
Ton Ca	-	/	oropharynx
Ton Ca	-	/	oropharynx
Ton Ca	-	/	oropharynx
Ton Ca	-	/	oropharynx
Larynx Ca	-	/	oropharynx
Larynx neoplasia	-	/	oropharynx
Larynx Ca	-	/	oropharynx
Larynx Ca	-	/	oropharynx
Larynx Ca	-	/	oropharynx
Larynx Ca	-	/	oropharynx
Larynx Ca	-	/	oropharynx
Larynx Ca	+++	100	larynx
Larynx Ca	+++	100	larynx
Oropharynx Ca	-	/	oropharynx
Oro-Hypopharynx Ca	+++	20	oropharynx *
Oropharynx Ca	-	/	oropharynx
Oropharynx Ca	-	/	oropharynx
Oropharynx Ca	-	/	oropharynx
Oropharynx Ca	+++	40	oropharynx *
Hypopharynx Ca	-	/	oropharynx
Hypopharynx Ca	-	/	oropharynx
Hypopharynx Ca	-	/	oropharynx
Larynx-Hypopharynx Ca	-	/	oropharynx
Tongue Ca	-	/	oropharynx
Tongue Ca	-	/	oropharynx
Tongue Ca	-	/	oropharynx
Tongue Ca	-	/	oropharynx
Tongue Ca	-	/	oropharynx
Tongue Ca	-	/	oropharynx
Tongue Ca	-	/	oropharynx
Mouth Ca	-	/	oropharynx
Mouth Ca	-	/	oropharynx
Mouth Ca	-	/	oropharynx
Mouth Ca	-	/	oropharynx
Vallecula Ca	++	20	oropharynx *
Vallecula Ca	-	/	oropharynx
Vocal cords Ca	-	/	oropharynx
Vocal cords Ca	-	/	oropharynx
Nasopharynx Ca	-	/	oropharynx
Alveolar ridge Ca	-	/	oropharynx
Uvula Ca	-	/	oropharynx
Nasal concha hyperplasia	-	/	nasal epithelium

Given are the intensity of K8 expression, diagnostic findings, percentages of positive cells within affected tissues, and localisation of the healthy mucosa.

-: no staining; +: weak; ++: intermediate, +++: strong. Chron. Tonsillitis: chronic tonsillitis; Ton Ca: tonsillar carcinoma.

*: CK8-positive cells represented either dysplatic or carcinoma cells within samples assessed.

Secondly, oral leukoplakia (n = 19), which in some cases represented pre-malignant lesions, were stained with K8 specific antibodies. In normal or hyperplastic leukoplakia (n = 9) 7 tissue specimens were negative for K8 (78%) and 2 specimens expressed K8 weakly in 10% and to intermediate levels in 5% of the cells, respectively (Table 2). Four out of six (66.7%) dysplastic leukoplakia expressed K8 to intermediate (++) or even strong (+++) levels in varying percentages of cells. Four leukoplakia samples actually represented carcinomas of small sizes (T1-2) and all expressed K8 to strong (+++) levels (Table 2).

**Table 2:** K8 expression in oral leukoplakia.

**LP**	**K8 INTENSITY**	**% OF TISSUE**	**DIAGNOSTIC FINDINGS**
**1**	-	/	normal
**9**	-	/	normal
**11**	-	/	normal
**5**	-	/	hyperplasia
**6**	-	/	hyperplasia
**7**	-	/	hyperplasia
**4**	-	/	hyperplasia
**14**	+	10	hyperplasia
**19**	++	5	hyperplasia
**2**	-	/	dysplasia (weak)
**12**	-	/	dysplasia
**8**	++	10	dysplasia
**10**	+++	90	dysplasia
**13**	++	5	dysplasia
**18**	+++	10	dysplasia
**17**	+++	10	SCC, G1
**15**	+++	20	T2, N0, Mx, G3
**16**	+++	20	T1, Nx, Mx, G1
**3**	+++	100	T1, G2-3

Given are the intensity of K8 expression, diagnostic findings accoding to TNM classification, and percentages of positive cells within affected tissues.

-: no staining; +: weak; ++: intermediate, +++: strong.

SCC: squamous cell carcinoma, no TNM status available; LP: leukoplakia.

Six lymph node metastases (LNM) from primary carcinomas of different origin expressed K8 to intermediate or strong levels and with a percentage range of positive cells from 5–100% (Table 3). Interestingly, three out of six LNM displayed a strong (+++) K8 expression in 100% of cells.

**Table 3:** K8 expression in lymph node metastases.

**LNM**	**K8 INTENSITY**	**% OF TISSUE**	**TNM/G**
CUP	++	5	G2
Soft palate Ca	++	5	T2/G2
CUP	++	70	G3
Adenocystic Ca	+++	100	G3
Tonsillar Ca	+++	100	T2/G2
Tongue Ca	+++	100	T2/G2

Given are the intensity of K8 expression, diagnostic findings of the cognate carcinoma accoding to TNM classification and grading (G), and percentages of positive cells within affected tissues.

-: no staining; +: weak; ++: intermediate, +++: strong.

Ca: carcinoma, CUP: carcinoma of unknown primary.

**Figure 2:**
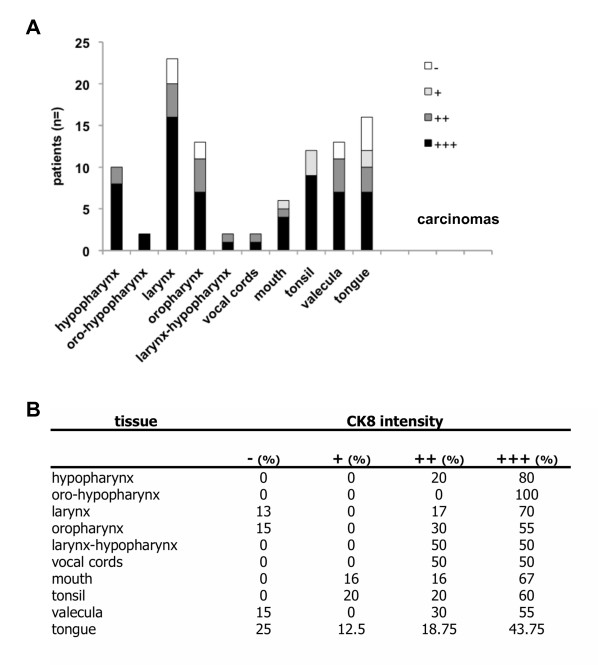
**K8 expression in head and neck squamous cell carcinomas.** (**A**) K8 was visualised by immunohistochemistry with specific antibodies in the indicated specimens of HNSCC, expression intensities are given as "no staining" (-), "weak staining" (+), "intermediate staining" (++), and "strong staining" (+++). (**B**) K8 staining intensities of tumour specimens are depicted as percentages of all specimens from one given origin.

In a last step, we assessed the expression of K8 in carcinomas (n = 102) of various localizations including hypopharynx (n = 10), oro-hypopharynx (n = 2), larynx (n = 23), oropharynx (n = 13), larynx-hypopharynx (n = 2), vocal cords (n = 2), mouth (n = 6), tonsil (n = 15), valecula (n = 13), and tongue (n = 16). Evaluation of K8 intensities in these malignancies demonstrated that intermediate and especially strong K8 expression was characteristic of head and neck carcinomas, independently of the sub-localization (Figure 2A). Negative or weak K8 expression accounted only for maximally 37.5% as seen for tongue carcinomas and was generally below 20% (mean value 11.65% ± 12). Oppositely, intermediate and strong K8 staining was seen in 62.5% to 100% of all cases (Figure 2B; mean value 88.25% ± 11.5).

### K8 expression in disseminated and tumour cells and metastases

**Figure 3:**
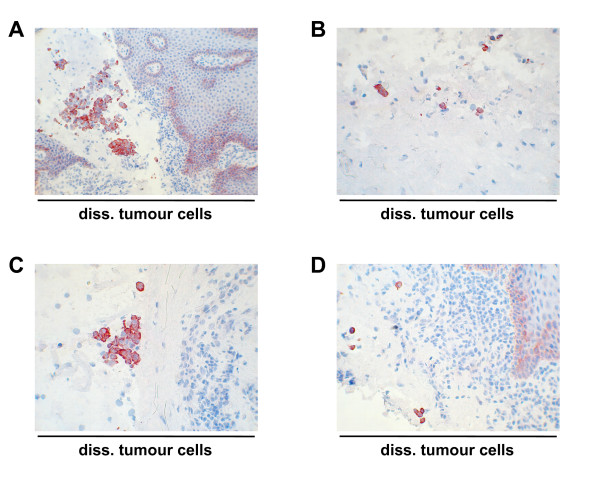
**K8 expression in detached tumour cells.** K8 was visualised by immunohistochemistry with specific antibodies in head and neck carcinoma specimens. Depicted are tumours cells that had detached from the primary tumour at the time of surgery and occurred as single cells or small islets of tumour cells (less than 30 cells).

**Figure 4:**
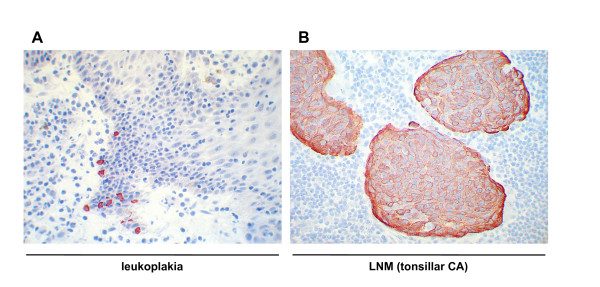
**K8 expression in leukoplakia and in lymph node metastases.** K8 was visualised by immunohistochemistry with specific antibodies at the protruding edge of dysplastic leukoplakia (*left panel*) and in lymph node metastases of a patient suffering from a tonsillar carcinoma (*right panel*).

Staining of various carcinoma samples with hematoxillin/eosin revealed the presence of tumour cells that had detached from the primary tumour into surrounding tissue at the time point of surgery. These disseminated tumour cells (DTCs) were in the close proximity of the bulk of carcinoma cells and were present either as single cells or as small nests composed of up to 15–30 cells. Immunohistochemical staining of these samples with antibodies specific for K8 strongly marked detached cells. Interestingly, K8 expression in DTCs was often superior to cells of the primary tumour suggesting a need for single cells to overexpress K8 (Figure 3). Noteworthy, detached and K8 positive cells were commonly not seen within densely packed infiltrates of immune cells but rather in loosened areas of the tissue specimens. In accordance with those findings, K8 positive cells with the highest expression levels often came to localize at the edge of tissue protruding into the submucosa, eventually generating a remarkable lining of K8^high ^cells, i.e. cells displaying strong K8 expression (Figure 4A). Since K8^high ^was a hallmark of detached carcinoma cells, we next analyzed its pattern in lymph node metastases of tonsillar carcinomas. Again K8^high ^was strictly confined to islets of metastatic tumour cells (Figure 4B).

## Discussion

Reliable markers for pre-malignant lesions retained their paramount importance, as they are believed to bring about significant improvements of patients' care and overall survival [27]. This notion was best exemplified by the use of prostate-specific antigen PSA for the early diagnosis of prostate carcinoma [28]. Clearly, the earlier a malignancy of the head and neck area is diagnosed, the better the prognosis for the patient [29]. Hence, detection of pre-malignant lesions is highly desirable as is the visualization of disseminated tumour cells, which are to be seen as founders of metastases [30-32]. With these prerequisites in mind it is interesting to retrieve from the present stuy that K8 (*i*) is absent in normal mucosa composed of squamous epithelium and in contrast to adenomatous epithelium, that (*ii*) K8 expression differentiates dysplastic lesions, carcinomas *in situ*, and small established carcinomas (*i.e. *T1-2) from normal tissue and hyperplastic lesions within oral leukoplakia, and finally that (*iii*) K8 thoroughly marks single detached tumour cells. These features and the *de novo *expression of qualify K8 as a worth candidate for the early detection of pre-malignant lesions, which might progress to overt malignancies with significantly enhanced probability [33], and for disseminated tumour cells in head and neck carcinomas. Additionally, K8 was released as a circulating marker from apoptotic non-small cell lung carcinoma cells as a full-length and proteolytic cleavage-resistant protein and might hence represent a valuable biomarker for head and neck malignancies [34].

From a mechanistic and molecular point of view, and with respect to the over-expression of K8 in cancer cells, two eventualities are to be envisaged. Firstly, disseminating and invading cells might require a reorganization of their cytoskeleton to improve motility and epithelial-to-mesenchymal transition (EMT), which could be warranted by keratins of simple epithelia as is K8. Data from Chu and colleagues disclosed an ability of K8 and K18 to foster the invasive potential when ectopically expressed in murine L fibroblasts [16]. Along this line, assessment of p38 kinase activity in disseminated cells appears expedient. K8 Ser^73 ^serves as a substrate for p38 kinase and phosphorylation at this position is crucial for the destabilization of intermediate filaments [3,5,35]. Reorganization, especially destabilization of intermediate filaments occurs under various physiological conditions such as mechanic stress, during epithelial cell homeostasis, exposure to chemicals (vanadate, ocadaic acid), and during mitosis. Common to all these processes is the recruitment of active p38 kinase to depolimerized keratin granules and phosphorylation of K8 at Ser^73 ^[5,11]. Hence, one may also envisage the simultaneous assessement of K8 expression and of Ser^73 ^phosphorylation as an additional surrogate marker for the mitotic index. A second scenario must be considered, in which K8 serves as a receptor for plasminogen and tPA at the plasma membrane of tumour cells [12-14]. By doing so, K8 will enhance the proteolytic activity at the plasma membrane and facilitate tissue remodelling and invasion. Such an eventuality is supported by data presented herein. Single tumour cells that were already detached from the main tumour at the time of sample asservation displayed highest K8 expression, which may foster proteolytic activity and invasion. Regulatory processes involved in the de novo expression of K8 in these tumour cells are poorly understood. Recent work by Lacina *et al. *disclosed a potential for non-malignant stroma cells, *i.e. *tumour-associated fibroblasts, to induce K8 expression on normal keratinocytes *in vitro *[36,37]. Thus, the tumour microenvironment needs to be seen as a strong modulator of protein cell surface expression of carcinoma cells. Clearly, both eventualities, namely remodelling of cell structures and improvement of the proteolytic appartus of carcinoma cells, are not mutually exclusive but may even be both instrumental in parallel.

The findings presented in this study of K8 expression are in accordance with and complementing previous data that demonstrated *de novo *synthesis of K8 in dysplastic lesions as well as in head neck carcinomas. However, these former studies were conducted in substantially smaller cohorts and without differentiation of tumour sub-localizations [25,38]. Subdividing head and neck tissues according to their precise localization revealed minor differences in the K8 expression profile. Hypopharynx, oropharynx, larynx, valecula, and vocal cords were characterized by > 85% of samples expressing intermediate to strong levels of K8. In contrast, carcinomas of the tongue were different since they displayed a more heterogeneous repartition with 37.5% of samples expressing no or weak levels of K8. Also, normal mucosa from the larynx was always positive for K8, pinpointing differences amongst healthy epithelia, too. Therefore, tongue and laryngeal carcinomas appear less amenable to a K8-based diagnosis.

Taken together our data qualify K8 as an excellent marker for head and neck carcinomas, lymph node metastases, and for tumour cells that have already detached from the primary tumour.

## Conclusion

The intermediate filament protein K8 is known as a tumour-associated antigen. We present a survey of K8 expression in head and neck epithelia that demonstrates the specific staining of K8 in pre-malignant and malignant tissue versus normal cells. Dysplastic and tumour cells expressed K8 to strong levels, as did disseminated tumour cells. Hence, K8 is an excellent marker for the visualisation and diagnosis of pre-malignancies and DTCs in the head and neck area.

## Abbreviations

*K8*: cytokeratin 8; *K18*: cytokeratin 18; *DTC*: disseminated tumour cell; *HNSCC*: head and neck squamous cell carcinoma; *MAP kinase*: mitogen-activated protein kinase; *PBS*: phosphate balance salt solution; *PKC*: protein kinase C; *RT*: room temperature.

## Competing interests

The authors declare that they have no competing interests.

## Authors' contributions

CM and AB were in charge of patients' recruitment, sample assessments, and study design. BM performed all stainings shown in the manuscript. OG wrote the manuscript, analysed the acquired data, and was responsible for the study design together with CM. All authors read and approved the final manuscript.

## Pre-publication history

The pre-publication history for this paper can be accessed here:


